# Combining Nimotuzumab With Chemotherapy for Patients With Locally Advanced and Intermediate-Stage Nasopharyngeal Cancer: A Retrospective Comparison Study Using Five-Year, Real-World Survival Data

**DOI:** 10.7759/cureus.48804

**Published:** 2023-11-14

**Authors:** Andhika Rachman, Sakinah Rahma Sari, Arie Munandar, Marlinda Adham, Susanna H Hutajulu

**Affiliations:** 1 Internal Medicine, Dr. Cipto Mangunkusumo Hospital - Faculty of Medicine Universitas Indonesia, Jakarta, IDN; 2 Hematology and Medical Oncology, Dr. Cipto Mangunkusumo General Hospital - Faculty of Medicine Universitas Indonesia, Jakarta, IDN; 3 Radiation Oncology, Dr. Cipto Mangunkusumo General Hospital - Faculty of Medicine Universitas Indonesia, Jakarta, IDN; 4 Otolaryngology - Head and Neck Surgery, Dr. Cipto Mangunkusumo General Hospital - Faculty of Medicine Universitas Indonesia, Jakarta, IDN; 5 Division of Hematology and Medical Oncology, Department of Internal Medicine, Dr. Sardjito General Hospital, Yogyakarta, IDN; 6 Division of Hematology and Medical Oncology, Department of Internal Medicine, Faculty of Medicine, Public Health, and Nursing, Universitas Gadjah Mada, Yogyakarta, IDN

**Keywords:** survival, prognosis, chemoradiation, anti-egfr, egfr, nimotuzumab, nasopharyngeal carcinoma

## Abstract

Background: Nasopharyngeal carcinoma (NPC) is the most prevalent geographically-specific head and neck cancer. Its incidence was high in the Asian population, especially in certain parts such as Southern China and South East Asia. Most patients with NPC are presented with intermediate-stage or locally advanced disease requiring chemoradiation as the primary treatment of choice. Epidermal Growth Factor Receptor (EGFR) was found overexpressed in most patients with NPC associated with poor prognosis making its inhibitor one of the most plausible treatment options in addition to chemoradiation. In EGFR-positive NPC patients, nimotuzumab, a humanized anti-EGFR monoclonal antibody will bind the extracellular domain of EGFR leading to tumor growth suppressions. This study’s objective was to assess the real-world clinical efficacy of nimotuzumab for patients with intermediate-stage and locally advanced NPC when in combination with concurrent chemoradiation.

Methods: This retrospective real-world study examined a sample of intermediate-stage and locally advanced NPC patients who were treated with or without adding nimotuzumab to concurrent chemoradiation at Dr. Cipto Mangunkusumo General Hospital in Indonesia from January 2009 to December 2017. The outcomes were patients’ real-world five-year overall survival (rwOS) and progression-free survival (rwPFS) compared using Kaplan-Meier analysis and Cox proportional hazard models adjusting for age, gender, comorbidities, clinical staging, staging based on Tumor status (T), staging based on Nodes status (N), and types of radiotherapy.

Results: A total of 407 patients were included in the analysis, 61 patients receiving concurrent nimotuzumab and chemoradiation and 346 patients receiving chemoradiation alone. Patients receiving concurrent nimotuzumab and chemoradiation tended to have less aggressive NPC than patients receiving chemoradiation alone. Multivariate-adjusted Cox models revealed that combining nimotuzumab with chemoradiation was associated with a statistically significant longer rwOS gain (hazard ratio (HR)=0.46 (95% CI: 0.26-0.82, p=0.008)) and a trend of longer rwPFS (hazard ratio (HR)=0.67 (95% CI: 0.41-1.09, p=0.109)) in comparison to chemoradiation alone.

Conclusion: In this retrospective real-world study, concurrent nimotuzumab and chemoradiation usage was associated with a significant overall survival benefit than chemoradiation alone for intermediate-stage and locally advanced NPC patients. Hence, adding nimotuzumab to patients’ chemoradiation should be considered in patients with intermediate-stage and locally advanced NPC.

## Introduction

Nasopharyngeal carcinoma is one of the most common types of cancer in the head and neck region, with a unique geographical distribution [1]. It is most prevalent in the Asian population. Indonesia has become the second country with the highest incidence after China, with an incidence rate of 5,7 per 100.000 cases in men and 1,9 per 100.000 cases in women. This number is much higher than the global incidence rate given that the cancer cases in Indonesia are not well-documented, indicating a possible higher number in reality [2].

Nasopharyngeal carcinoma is stratified into several stages according to the Tumor, Node, Metastasis (TNM) classification system. The treatment approach was based on what stages patients presented when admitted. Currently, there are various treatment modalities for nasopharyngeal carcinoma, such as radiotherapy, chemotherapy, targeted therapy, or surgery, with the former being more commonly used than the latter [3]. Even though nasopharyngeal carcinoma is radiosensitive, most patients who presented with locally advanced disease required additional chemotherapy in their treatment regimens [4-6]. The combination of radiotherapy and chemotherapy has improved the overall survival in patients with nasopharyngeal carcinoma. However, about 25% of patients were facing a treatment failure, thus an alternate treatment modality has to be considered in addition to chemoradiation [7]. This is becoming more important considering the adverse events that patients experience after chemoradiation [8]. One to be acknowledged is a targeted therapy in which an antibody acts toward a specific protein involved in tumorigenesis. Examples of targeted therapy are antibodies against epidermal growth factor receptor (EGFR) and vascular endothelial growth factor (VEGF) [9].

The overexpression of EGFR in nasopharyngeal tumor cells enables its function as a treatment modality a legitimate option for patients with intermediate-stage and locally advanced disease [9]. Nimotuzumab, as one of the monoclonal antibodies against EGFR, has been found to have an effect on reducing tumor volumes despite its role in increasing overall survival in patients with locally advanced nasopharyngeal carcinoma (NPC) while in combination with other treatment regimens is still debatable. Previously, a phase III randomized clinical trial by Kong et al., involving 135 NPC patients, revealed that concurrent nimotuzumab with intensity-modulated radiation therapy (IMRT) following TPF-based induction chemotherapy in comparison with concurrent chemoradiation with Cisplatin had a similar effect with lower toxicities [10]. Hence, this observational study was conducted to evaluate the real-world data of adding nimotuzumab to other treatment regimens and its effect on patients with intermediate-stage and locally advanced nasopharyngeal carcinoma in clinical practice.

This article’s abstract will be presented as a poster display at ESMO Asia Congress 2023 on December 1-3, 2023.

## Materials and methods

Data source

This study was approved by the Faculty of Medicine, University of Indonesia’s ethical committee with approval number KET-456/UN2.F1/ETIK/PPM.00.02/2023. The data used in this study were obtained from electronic and printed medical records at the Department of Ear, Nose, and Throat (ENT), the Department of Radiation Oncology, and the Department of Internal Medicine, Dr. Cipto Manungkusumo General Hospital. Data regarding patients’ status not documented in the medical record was extracted from the civil registry.

Patient selection

A total of 439 patients admitted with nasopharyngeal carcinoma from January 2009 to December 2017 were evaluated retrospectively. The inclusion criteria were patients diagnosed with nasopharyngeal carcinoma from all age groups who were either receiving nimotuzumab or chemoradiation. Patients with stage I nasopharyngeal carcinoma, metastatic disease, and patients with unknown staging were excluded from the study. Data extraction was conducted from March 9, 2023, to March 31, 2023. From the data collected, 84 out of 439 patients were receiving nimotuzumab. The remaining 355 patients were treated with chemoradiation, which was the control of this study. All the data taken were then tabulated in a Microsoft Excel (Microsoft Corporation, Redmond, WA) table.

Study endpoint

The primary outcome assessed in this study was five-year real-world overall survival (rwOS) in patients receiving nimotuzumab or control. Another outcome, such as five-year real-world progression-free survival (rwPFS) was assessed. Five-year rwOS was defined as patients’ survival from any cause of death in five years after the initiation of patients’ therapy. Meanwhile, five-year rwPFS was defined as patients’ survival from their disease progression which includes tumor growth and spread. Patients’ progression was determined from the follow-up radiological examination results taken from patients' medical records. The start index date was the first day that patients received treatment: either nimotuzumab or chemotherapy. Patients without recorded death or loss of follow-up were censored.

Data analysis

This study performed the analysis using SPSS version 27.0 (IBM Corp., Armonk, NY). The primary outcome of this study was presented as Kaplan-Meier survival curves and the Cox proportional hazard analysis. Other variables regarding baseline characteristics between the two groups were evaluated using crosstabs, followed by chi-square tests for categorical variables and the Wilcoxon rank sums test for continuous variables. The effect of different treatment care for patients on rwOS and rwPFS was tested using the multivariate analysis with adjustment for gender, age, comorbidities, clinical staging, staging based on Tumor status (T), staging based on Nodes status (N), and types of radiotherapy.

## Results

Patients’ socio-demography

Among the 439 screened patients, 2 patients were receiving nimotuzumab with a diagnosis of stage I nasopharyngeal carcinoma and 2 patients had metastatic disease; thus they were excluded. A total of 11 patients, 9 patients receiving nimotuzumab and 2 receiving control, were also excluded due to unknown staging based on their medical records. Patients with unknown records of radiotherapy modalities were excluded, 10 patients from the nimotuzumab receiving group and 7 patients from the control group. Therefore, the remaining 407 patients were eligible for further analysis. 

The radiotherapy (RT) regimens recorded were 2-dimensional RT, 3-dimensional conformal RT, and intensity-modulated radiation therapy (IMRT). The RT doses were equivalent to 70 Gy for gross tumor and nodes, 60 Gy for high-risk areas, and 50 Gy for elective nodes. The chemotherapy regimens included were cisplatin, 5FU, carboplatin, paclitaxel, and capecitabine. The dose of nimotuzumab in the interventional group was 200 mg weekly.

In this study, it was revealed that the proportion of sex distribution was equal in both the nimotuzumab-receiving group and the control group (p = 0.162). The age of patients was also similar in both groups (p = 0.276). Even though both groups, the majority of patients were classified as WHO type 3 by histopathologic findings, with 73.8% in patients receiving nimotuzumab and 87.6% in patients receiving the control, the distribution was significantly uneven (p = 0.00).

In both groups, most patients were diagnosed in the late stage (clinical stage IVA) even though the difference was enormous (45.9% vs 81.8%, p = 0.00). Comorbidities such as cardiovascular disease, diabetes mellitus, renal disease, and liver disease were found more in patients who didn’t receive nimotuzumab (44.2% vs 14.1%, p = 0.00). Adjusted comparisons between a combination with nimotuzumab and chemoradiation alone were conducted using multivariate Cox models, controlling for patient baseline characteristics, including age, gender, comorbidities, clinical staging, staging based on Tumor status (T), staging based on Node status (N), and types of radiotherapy. More about patients’ characteristics included in this study is shown in Table 1.

**Table 1:** Patients’ characteristics T = Tumor, N = Nodes

	Nimotuzumab	Control	P value
Sex			0.162
Female	12 (19.7%)	101 (29.2%)	
Male	49 (80.3%)	245 (70.8%)	
Age	16-76 (47.4)	18-77 (45.9)	0.276
WHO Classification			0.001
Type 1	5 (8.2%)	2 (0.6%)	
Type 2	4 (6.6%)	25 (7.2%)	
Type 3	45 (73.8%)	303 (87.6%)	
N/A	7 (11.5%)	16 (4.6%)	
Clinical Stages			0.000
II	17 (27.9%)	3 (0.9%)	
III	16 (26.2%)	60 (17.3%)	
IVA	28 (45.9%)	283 (81.8%)	
TNM Staging			0.000
T1	4 (6.6%)	10 (2.9%)	
T2	24 (39.3%)	47 (13.6%)	
T3	11 (18.0%)	42 (12.1%)	
T4	22 (36.1%)	247 (71.4%)	
N0	14 (23.0%)	27 (7.8%)	
N1	13 (21.3%)	51 (14.7%)	
N2	25 (41.0%)	162 (46.8%)	
N3	9 (14.8%)	106 (30.6%)	
Comorbidities			0.000
With Comorbidities	9 (14.8%)	152 (43.9%)	
Without Comorbidity	52 (85.2%)	194 (56.1%)	
Types of Radiotherapy			0.001
2D	18 (29.5%)	187 (54.0%)	
3D	12 (19.7%)	37 (10.7%)	
IMRT	31 (50.8%)	122 (35.3%)	

Adverse reactions

Adverse reactions were reported in 32.8% of patients receiving nimotuzumab (Table 2). The adverse reactions were mucositis experienced by 24.6% of patients, xerostomia in 11.5% of patients, hyperpigmentation in 18% of patients, and nausea in 1.6% of patients.

**Table 2:** Adverse reactions in patients receiving nimotuzumab

	Mucositis	Xerostomia	Hyperpigmentation	Nausea
Number of Patients	15 (24.6%)	7 (11.5%)	11 (18.0%)	1 (1.6%)

Survival analysis

**Figure 1:**
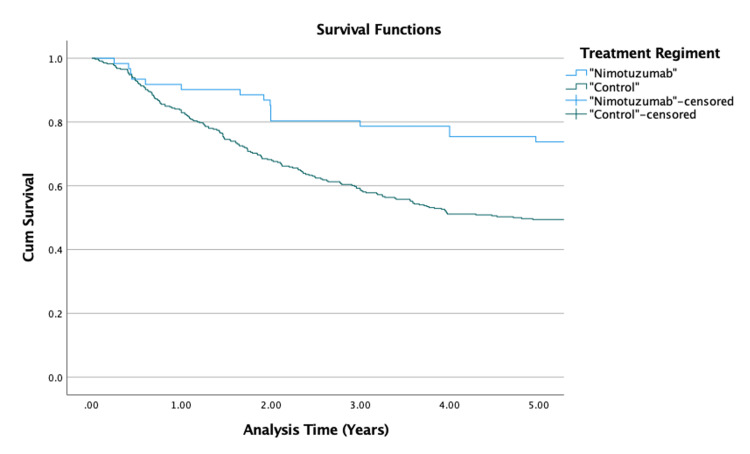
Kaplan-Meier survival curves for five-year rwOS rwOS: real-world overall survival

Kaplan-Meier survival analysis (Figure 1) was used to present the five-year rwOS curves for the two groups. The five-year overall survival of patients with NPC in Dr. Cipto Mangunkusumo General Hospital was 53.1%. The results revealed significantly better five-year rwOS among patients receiving nimotuzumab in their treatment regimens than the patients receiving standard regimen (73.8% vs 49.4%, p = 0.01). The mean survival in all of the patients was estimated as 8.4 years. The mean survival time in patients receiving nimotuzumab was estimated as 11.12 (95% CI: 9.731-12.509) years while the other group was estimated as 6.23 (95% CI: 5.748-6.716) years.

**Figure 2:**
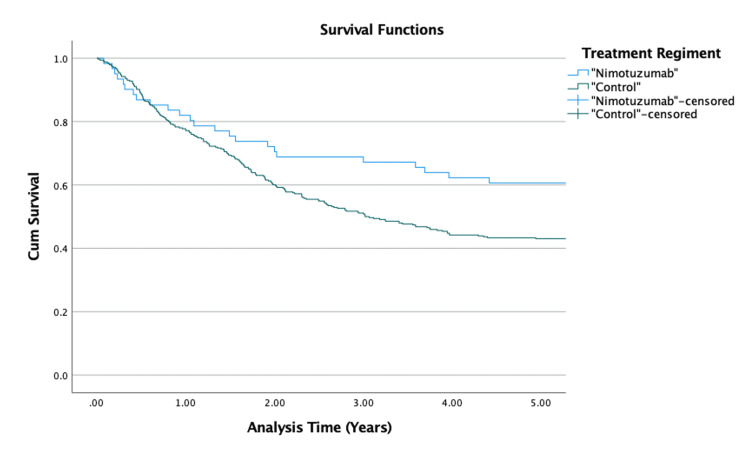
Kaplan-Meier survival curves for five-year rwPFS rwPFS: real-world progression-free survival

Patients’ rwPFS was also analyzed using Kaplan-Meier survival analysis (Figure 2). About 60.7% of patients in the nimotuzumab-receiving group survived the five-year disease progression while in the control group, about 42.2% of patients survived. The unadjusted result was significantly different (p = 0.02). The mean progression-free survival in the nimotuzumab-receiving group was 9.181 (95% CI: 7.612-10.750) years while in control was 5.425 (95% CI: 4.946-5.904) years.

Confounding factors

Confounding factors, such as gender, age, comorbidities, clinical staging, staging based on Tumor status (T), staging based on Node status (N), and type of radiotherapy were adjusted using multivariate analysis. With such adjustment, rwOS was significantly longer for nimotuzumab-based combined radiotherapy patients compared to chemoradiation-alone patients (adjusted hazards ratio (aHR) = 0.46 (95% CI: 0.25-0.81), p = 0.008).

Meanwhile, multivariate-adjusted Cox analysis showed that adding nimotuzumab did not significantly improve patients’ five-year rwPFS with an aHR of 0.67 ((95% CI: 0.41-1.09), p = 0.109). The result of multivariate analysis was shown in Table 3 while the comparison of nimotuzumab vs control before and after adjustment was shown in Table 4.

**Table 3:** Multivariate analysis Patients receiving chemoradiation (control) formed the reference group. aHR: adjusted hazards ratio

Indicators (Reference)	Overall Survival (rwOS)			Progression-Free Survival (rwPFS)		
	aHR	95% CI	P value	aHR	95% CI	P value
nimotuzumab vs control^1^	0.456	0.255-0.815	0.008	0.666	0.405-1.094	0.109
gender	0.953	0.692-1.312	0.768	0.966	0.716-1.303	0.820
age	1.015	1.002-1.028	0.020	1.008	0.996-1.020	0.197
clinical staging			0.655			0.686
Stage III	0.680	0.222-2.087	0.500	0.922	0.358-2.372	0.866
Stage IVA	0.894	0.286-2.799	0.848	1.214	0.462-3.189	0.694
tumor stage			0.282			0.752
T2	2.851	0.866-9.390	0.085	1.357	0.601-3.062	0.462
T3	2.236	0.646-7.744	0.204	1.045	0.435-2.514	0.921
T4	3.035	0.909-10.130	0.071	1.321	0.568-3.070	0.518
nodes stage			0.043			0.359
N1	1.459	0.729-2.920	0.286	1.097	0.619-1.947	0.750
N2	1.780	0.949-3.337	0.072	1.097	0.651-1.848	0.729
N3	2.414	1.241-4.699	0.009	1.473	0.841-2.580	0.175
comorbidities	0.847	0.620-1.156	0.296	1.002	0.749-1.340	0.988
radiation therapy			0.168			0.496
3D	1.142	0.710-1.836	0.585	1.140	0.741-1.753	0.551
IMRT	1.361	0.988-1.875	0.059	1.192	0.886-1.604	0.247

**Table 4:** Comparison of nimotuzumab vs control before and after adjustment 1. Patients receiving chemoradiation (control) formed the reference group. 2. The model was adjusted for the variables: age, gender, comorbidities, clinical staging, staging based on Tumor (T), staging based on Nodes (N), and types of radiotherapy.

Nimotuzumab vs Control^1^	Overall Survival			Progression-Free Survival		
	HR	95% CI	P value	HR	95% CI	P value
Unadjusted	0.433	0.259-0.723	0.001	0.603	0.395-0.921	0.019
Adjusted^2^	0.456	0.255-0.815	0.008	0.666	0.405-1.094	0.109

## Discussion

Nimotuzumab for patients with NPC

The five-year rwOS and five-year rwPFS between the nimotuzumab receiving group and the control group in our study were different. Previous studies have shown that nimotuzumab improved patients’ overall remission rate and overall survival when in combination with chemoradiation. A meta-analysis conducted by Yuan et al. revealed that a combination of nimotuzumab and RT/chemoradiation had a better overall remission rate (ORR) of the primary tumor than a combination of cetuximab with RT/chemoradiation (ORR = 3.21 (95% CI: 1.97-5.21)) or RT/chemoradiation alone (ORR = 4.11 (95% CI: 2.43-6.94)). Their results also showed that nimotuzumab had a trend of longer three-year OS than a combination of cetuximab with RT/chemoradiation (OS rate = 4.22 (95% CI: 1.61-11.05)) or RT/chemoradiation alone (OS rate = 2.05 (95% CI: 0.62-6.76)) [11]. A study by Liu et al. evaluating nimotuzumab's efficacy in combination with chemoradiation in 42 NPC patients reported that the ORR was 100% and the two-year OS was 96.6% [12]. Another study conducted by Fangzheng et al. revealed a satisfactory result with a three-year OS of 86.8% [13]. Whilst the ORR was not analyzed, our study affirmed that patients receiving nimotuzumab in their chemoradiation had better OS than patients receiving chemoradiation alone gained (hazard ratio (HR)=0.46 (95% CI: 0.26-0.82, p=0.008)). In comparison with the study by Liu et al. and Wang et al., we reported lower OS (73.8%) but with a longer period of follow-up time and a larger amount of patients. Consistent results of better OS in patients receiving nimotuzumab were hypothetically related to its mechanism in which nimotuzumab will enhance Cisplatin's effect by involving the EGFR/AKT activation. Thus, adding nimotuzumab to chemoradiation could benefit patients with NPC by increasing the apoptotic effect of chemotherapy [14].

In spite of a better five-year rwOS in patients receiving nimotuzumab, the five-year rwPFS was not significantly different even though the trend was better in the nimotuzumab-receiving group (HR=0.67 (95% CI: 0.41-1.09, p=0.109)). Regardless of the non-significant rwPFS, our result showed that adding nimotuzumab to patients' chemoradiation improved patients' rwPFS by 18.5%. Our study was in accordance with a larger propensity score-matched analysis by Zhi-Qiang et al., which revealed a slightly better five-year PFS in the nimotuzumab receiving group (79.96% vs 77.99%, p=0.117) involving 730 patients with NPC [15]. A phase II clinical trial by Huang et al. reported a better PFS of 83.5% [16]. Another propensity score-matched analysis conducted by Yao et al. showed that the five-year PFS was significantly better in patients receiving nimotuzumab than patients receiving chemoradiation alone even after adjustment (aHR=0.38 (95% CI, 0.11-0.89, p=0.041)) [17]. In comparison with the study by Huang et al. and the study by Yao et al., our study involved a larger amount of patients and a longer period of follow-up time. An essential point to take note of was that the disease progression was evaluated with a radiological examination, either a follow-up CT scan or MRI after a month, two months, and three months while the gold standard was a follow-up nasopharyngeal MRI test after 12 weeks. With various modalities in evaluating patients' progression, the five-year rwPFS should be interpreted carefully.

Aside from its benefit for patients' survival, our study revealed that patients receiving nimotuzumab had fewer toxicities. Some of the minimal adverse reactions reported were mucositis, hyperpigmentation, xerostomia, and nausea experienced by 32.8% of patients receiving nimotuzumab and were considered tolerable. This result was consistent with a study by Chen et al. who used cetuximab and nimotuzumab in combination with radiotherapy for locally advanced nasopharyngeal carcinoma patients showed that patients receiving cetuximab in their care had a higher incidence of mucositis than patients treated with nimotuzumab (87% vs 15%) [18]. The mechanism was postulated in a study by Takeda et al., stating that nimotuzumab had a different binding manner than other antibodies against EGFR. Dissimilar to cetuximab, which binds to cells with lower EGFR expression levels in a monovalent manner, nimotuzumab requires a divalent binding [19]. As a consequence, in cells with lower EGFR expression levels, such as skin, nimotuzumab will cause fewer adverse reactions.

Most of the proposed confounding factors, such as gender, clinical stage, staging based on Tumor status (T), comorbidities, and types of radiotherapy, had no significant effect on the five-year rwOS and rwPFS. Meanwhile, the result showed that older age slightly affected patients' five-year rwOS (aHR=1.015 (95% CI: 1.002-1.028, p=0.02)) and a non-significant effect in five-year rwPFS (aHR=1.008 (95% CI: 0.996-1.020, p=0.197)). Similarly, staging based on Nodes status (N), especially patients with N3, affected patients' five-year rwOS significantly (aHR=2.414 (95% CI: 1.241-4.699, p=0.009)) and a trend of worse rwPFS (aHR=1.473 (95% CI: 0.841-2.580, p=0.175). We omitted histological results based on WHO types from the Cox proportional hazard model due to its unrecorded data in some patients (N/A category). According to a study by Siti-Azrin et al., the WHO classification type did not significantly affect patients' prognosis [20]. Therefore, excluding the aforementioned variable should not interfere with our results.

Limitation

This study was conducted retrospectively, which could lead to sample size and selection bias. Being retrospective, it is inherently limited by the accuracy of historical medical records and potential confounders not accounted for in the analysis. Another essential fact concerning the unequal number of patients between the two groups should be taken into consideration while interpreting our results. In our study, data regarding patients' EBV DNA levels, which can affect patients' prognosis, was not evaluated. Lastly, this study did not compare nimotuzumab with other EGFR inhibitors directly, which could have provided clearer pictures of its relative efficacy.

## Conclusions

In summary, despite the above-mentioned limitations, this study provides a reference value in regard to nimotuzumab utilization for patients with intermediate-stage and locally advanced NPC. In this retrospective, real-world study of intermediate-stage and locally advanced NPC patients, concurrent nimotuzumab and chemoradiation usage was associated with a significant overall survival benefit than chemoradiation alone. Hence, the combination of nimotuzumab and chemoradiation should be considered in patients with intermediate-stage and locally advanced NPC. We recommend the conduction of larger, prospective, multi-center trials with balanced patients between the interventional and control groups in the future.
